# Editorial: The Role of Psychological Capital in Entrepreneurial Contexts

**DOI:** 10.3389/fpsyg.2020.582133

**Published:** 2020-12-08

**Authors:** Fu-Sheng Tsai, Karen M. Leonard, Shalini Srivastava

**Affiliations:** ^1^North China University of Water Resources and Electric Power, Zhengzhou, China; ^2^Department of Business Administration, Cheng Shiu University, Kaohsiung, Taiwan; ^3^Center for Environmental Toxin and Emerging-Contaminant Research, Cheng Shiu University, Kaohsiung, Taiwan; ^4^Super Micro Mass Research and Technology Center, Cheng Shiu University, Kaohsiung, Taiwan; ^5^College of Business, University of Arkansas Little Rock, Little Rock, AR, United States; ^6^Jaipuria Institute of Management, Lucknow, India

**Keywords:** psychological capital, entrepreneurship, future studies, Research Topic, context

## Introduction

Psychological capital is a central topic in academic research on positive organizational psychology and behavior. Psychological capital is defined as an individual person's psychological capacity to handle and to respond to challenging situations. Self-efficacy, optimism, hope, and resiliency are the keystones of psychological capital. In the working environment, it has been proven as one vital factor for organizational and managerial performance. Important research questions related to psychological capital include its relationships to employee attitudes, behaviors, performance, as well as methodological issues (e.g., measurement) for researching this construct. For example, research has revealed that psychological capital helps prevent undesirable working attitude (e.g., cynicism) and behaviors (e.g., deviation).

As the literature and practical importance of psychological capital is growing^1^, more studies are needed for more fine-grained knowledge accumulation and advancement. For example, studies could investigate psychological capital's essence, functions, and implications in different contexts. For example, what constitutes an individual's psychological capital, and how it differs between for-profit and non-profit organizations. Another instance, startup new ventures and mature enterprises might require different kind of psychological capital and functionalities. Thus, psychological capital in an entrepreneurial context, as a refined construct, is strongly demanded during the complex processing of entrepreneurial activities for the new venture success.

Furthermore, interdisciplinary research between psychological capital and other scientific fields may help innovative lines of literature and thus broaden the influences of psychological capital studies in society. For example, what is the influences of new-generation technology on psychological capital? Can psychological capital be considered beyond Organizational Behavior and Human Resources and be connected to Strategic Management field of research? Should psychological capital be considered when organizations strategically choose strategic alliance partner?

The papers in this Research Topic represented new thoughts of researching PsyCap in a special context of entrepreneurship. In this section, we briefly review and criticize those scholarly pieces, in order to stimulate more reflections for the future studies. This review article utilizes a nomological taxonomy to discuss the papers published in the Research Topic, grounding one two theoretical axes—entrepreneurial activity (i.e., creativity, innovation, entrepreneurship, and ultra-entrepreneurial activities) and rationales (i.e., psychological, sociological, economic, political/institutional). To understand a research stream, both axes are important to sketch clearly the picture of literature structure, with the activity axis represent a process view of the studies while the rationale axis represents logical foundations that facilitates interpretation of the papers discussed.

## Research Frontiers and Imagination for Future Studies

In this section, we review specifically and discuss broadly the articles published in chronical order (their publication date). Bockorny and Youssef-Morgan examined the relationship between entrepreneurs' courage, psychological capital, and life satisfaction. Results show that entrepreneurs' courage is related to their life satisfaction, and that psychological capital fully mediates the relationship between courage and life satisfaction. This study took a courageous step ahead in the field of Entrepreneurship by empirically investigating the construct of entrepreneur's courage. In our nomological taxonomy (see Table 1), this study is in the category that concerns beyond entrepreneurial activities (i.e., ultra-entrepreneurial) from the perspective of an entrepreneur's social life. The rationale behind the theoretical construction is interwoven by PsyCap and sociological thoughts. With this study as an implicative first step, we encourage future studies to empirically examine the relationships of entrepreneurial courage with other entrepreneurship-related antecedents and outcomes, to enrich the impact of this construct in the knowledge domain of Entrepreneurship.

**Table 1:** Nomological taxonomy.

**Entrepreneurship** **Innovation** **Creativity** **Ultra-entrepreneurial** ****	**(Chen and Wu; Wang et al.)** **(Di Fabio and Duradoni; Lee and Yang; Tang et al.)** **(Li et al.)** **(Modesti et al.)**	**(Guo, Lu et al.; Kong F. et al.; Tang and Shao)** **(Chu et al.)** **(Kerksieck et al.)** **(Bockorny and Youssef-Morgan)**	**[Tang (b)]** **[Tang (a)]** **(Chen and Pan)** **(Xu et al.; Zhao et al.)**	**(Guo, Liu et al.)** **(Fang et al.)** **(Wu et al.)** **(Ren et al.)**
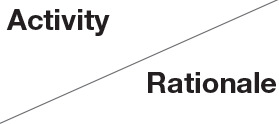	**Psychological**	**Social**	**Economic/** **Organizational**	**Political**

*By entrepreneurship we included a wider definitional scope to include general (economic) entrepreneurship, social entrepreneurship, institutional entrepreneurship, and so on.

Wu et al. worked creatively on the topic of “How Machiavellianism, Psychopathy, and Narcissism Affect Sustainable Entrepreneurial Orientation: The Moderating Effect of Psychological Resilience.” In many aspect, we could judge this study as very innovative for its cross-disciplinary nature. The major thesis of this study is that sustainable entrepreneurship relies on careful understanding of the micro-dynamics of the relationships between personality, entrepreneurial orientation, and psychological resilience. Therefore, relationships between the “dark triad” (Machiavellianism, psychopathy, and narcissism), psychological resilience, and sustainable entrepreneurial orientation were examined through a survey study. The results showed that the three traits influenced resilience and sustainable entrepreneurship orientation differently, implying that personality traits and psychological dynamics are complex and simultaneously functional and dysfunctional in practices. This study showed that when studying entrepreneurs as individual persons, psychiatric perspective, methods, and rationales could expand the research landscape, which is just right for our suggestion for expanded avenue for future studies.

Kong F.-Z. et al. studied another interesting group of workers (i.e., new-generation farmers) on their work-life quality and entrepreneurship will. As new-generation farmers now become important workforce and economic contributors in many cities, their entrepreneurial activities now become central imperatives. Inherently, this study focuses on entrepreneurship from a sociological lens. The authors found that “… farmers have a relatively low cognition level of their quality of work life, and their interpersonal relationship, work characteristics, material security, and family demands have significant effects on their entrepreneurship will.” Such finding not only offered a clear self-understanding for new-generation famers in China or in similar developing economies, but it also offered a solid reference for agribusiness policy makers.

Di Fabio and Duradoni have done a really interesting job in exploring a potentially to-be-widely-used construct [i.e., intrapreneurial self-capital (ISC)], which is faithfully defined as “as a possible primary preventive resource to effectively deal with the complexity of the current entrepreneurial environment.” Further, they distinguish this construct from PsyCap in many significant aspects, then connect it to innovative behaviors. Expectably, there would be a considerable future studies apply this construct into a wider scope of research.

Chen and Pan contributed by testing the influences of developmental job challenges (DJC) on venture performance through the mediated moderation of entrepreneurial action learning (EAL) and entrepreneurial experience. This work investigates on the action learning as entrepreneurial workers' creative activity from Organizational Behavior perspective. On the basis of improved methodology, future studies could follow their ideas and pay more attention to the antecedents and consequences in the context of entrepreneurship.

Wang et al. in their exploratory though descriptive analyses bravely tried the possibility of integrating the PsyCap into the framework of entrepreneurial intellectual capital. In addition to propose that the psychological capital, human capital, and relational capital are all necessary and representative capitals of entrepreneurs for new venture success, based on a pile of documentary data of real-life entrepreneur stories. Through this study, an attempt of formalization of psychological capital in entrepreneurial contexts (which is the central theme of this Research Topic) has been confirmed. Future studies should try to bring more empirical examinations for further clarifying the differentiated effects of different types of intellectual capital possessed by entrepreneurs or entrepreneurial teams.

In Guo, Lu et al. paper, the PsyCap is treated as a theoretical perspective that could explain the effect of entrepreneurial team's background structure and their strategic decision-making (here, investment decisions). This is an innovative trial, for PsyCap is usually adopted in scholarly research as a confirmed construct. By leveling up the PsyCap as the *collective* PsyCap, the authors found that PsyCap well-explained the examined relationships proposed in their research framework. They reported that “…the proportion of entrepreneurial team with the technological background is positively related to R&D expenditure and negatively related to marketing expenditure. On the contrary, we also find that the proportion of entrepreneurial team with a marketing background is a negative correlation with R&D expenditure and positive correlation with marketing expenditure.” Background structure can be interpreted as the social structure of an entrepreneurial group, and rationally of course, could generate impact on a state of psychological capital shared by the entrepreneurs. With this study, a new avenue of research might be opened, though, future studies are encouraged to test such thesis empirically by including the PsyCap as a testable construct.

Another interesting and important work, done by Chen and Wu, has given us a lot of imagination in a very broad academic landscape. Their paper argued that the PsyCap of the *public in a society* is the foundation of food safety governance. To a certain degree, such conceptualization is similar with the Guo, Liu et al. work above, in that the level of measurement and analysis of the PsyCap is up onto a collective-level. The difference is that the level represented by the “collective” in the Chen and Wu article is even higher (i.e., PsyCap shared by the public in a society). The linkage between the micro-macro levels are so courageous that this article sheds light on the psychological micro-foundation shared by a public collective and its influences on a public affair. Two scholars argued that such psychological micro-foundation could enable their called social governance for food safety that is co-created by the public who share the PsyCap. Furthermore, they seriously discussed the distinctive effects of the four elements of the PsyCap (i.e., self-efficacy, optimism, hope, and resiliency) in line with the co-governance of food safety. As was firmly proposed, “… that great success in food safety co-governance would be realized if the government, industry, and society nurture positive psychological capital.” More specifically, companies and government in food area could exercise social co-governance jointly by working hard “to appeal to the emotions of food companies and social actors to ensure self-efficacy toward food safety,” to “inspire hope by setting food safety goals and plans to achieve them,” to “motivate food companies and promote self-efficacy in co-governance efforts,” and to “utilize social persuasion to improve the engagement of social actors in food safety regulations.” In nature, this work's essence and contributions is perfectly entrepreneurial and institutional, in that it gave hints on the possibility of looking into public affairs as research issues/questions from the PsyCap both as a construct and as a theoretical lens. Also, they applied the concept of PsyCap to a new and vital field of food safety governance. In such vein, we would look sincerely forward to seeing an empirical examination based on this solid theoretical ground.

Tang and Shao conceptualized the influences of PsyCap possessed by a sub-group of workforce (inter-organizational Management Information System developers) in an organization on successful social innovation, by arguing that the PsyCap could improve the workforce's working effectiveness beyond the boundary of single organization. In other words, they argued that PsyCap shared by the MIS developer could improve their effectiveness and volunteering intentions of inter-organizational coordination and collaboration, and then result in a successful development of new MIS system that stimulate social innovation (or a successful social innovation embodied as a generation of an inter-organizational MIS). This paper pushes the PsyCap research by step ping into the Technology Management field and sketches the visionary future that how PsyCap is important to generate impact on social innovation via social innovation developers and technologies. This justifies the reason why the paper is located in the intersection of innovation activity from the Economic/Organizational perspective.

A comparable work that involves PsyCap in inter-organizational affair is completed by Chu et al. in a development project context of public-private collaborations on the innovative accounting revolution—green accounting. The article argued that “through individual and/or collective psychological states affected by demographic attributes, top managers shape the corporate culture and determine the overall strategic directions of an organization.” Based on this premise, this article discussed “the role psychological capital plays in the relationship between top manager attributes and the effectiveness of green accounting practices adoption in a public-private partnership (PPP) context.” Through related but distinctive paths of logical explanations, this paper is comparable with the work of Tang and Shao and the Guo, Liu et al., in that they all shared interests in researching PsyCap's impact on a group of people with very clear job goals that go beyond organizational boundaries.

Li et al. work on the role of PsyCap in the humorous leadership-employee creativity relation brings us a sense of muse in the conduction of organizational studies. This study used the concept of PsyCap to answer the question of seeking for a theoretical mechanism that well justifies the effect of humorous leadership on creativity. Through a rigor methodological design and data set of supervisor-subordinate dyads, a partial mediation effect of the PsyCap is confirmed. The results if of great practical reference, since leadership is an issue that is always at the core of organizational studies. For the current Research Topic, the results generate good practical implications for entrepreneurs as leaders in an uncertain organizational development stage.

Fang et al. worked on the mediating role of PsyCap in the relationship between inclusive leadership and employees' innovative behaviors. Clearly, this paper identified the PsyCap as the psychological theoretical mechanism that could turn the impact of inclusive leadership into realized innovative behaviors of employees. Providing practical implications and guidance, this study demonstrated that PsyCap is a functional factor that could bring the effort of leaders into employees' minds and stimulate their constructive behaviors toward the whole organization. One the other hand, however, this result reminds again, as many other leadership studies did, that politically or tactically, the choice of leadership styles can be a tool that manipulate an employee's mindset and his/her behavior.

Lee and Yang contributed by linking the literature of PsyCap to a farther research playground of Marketing. It is with no surprise that a mental mechanism or resource like the PsyCap would be chosen to be discussed of its potential with the works in Marketing, since psychology is also a upstream literature of Marketing and (especially) consumer research. Nonetheless, by detailed articulation in their opinion on the four dimensions of the PsyCap, this article still brought out sight to a good imagination for future studies shine at the intersection of Psychology and Marketing.

Everyday working is embedded in everyday living. Ren et al. paper is worth of attentions from the policy makers and maybe all non-academic readers who care about housing as a livelihood issue. With entrepreneurial nature, housing is an imperative that public government needs to take care of innovatively with the dramatically changing environments. Housing is also a critical ultra-entrepreneurial factors that might explicitly or implicitly affect employees' minds and conducts in an entrepreneurial context. So, as they noted, the study “identified three critical housing-related factors (HRFs) as moderators of the relationships between employees' psychological capital (PsyCap) and job embeddedness (JE) in China's entrepreneurial environment…” and by doing so the “results contribute to the PsyCap and JE literature by incorporating housing as an extrinsic life-aspect factor that might affect employees' psychological state and thus their retention in works.”

Kerksieck et al. zoomed in the relational details between the personal (PsyCap) and social (job) resources at work, with innovative consideration of job crafting as the catalyst of the dynamics between the two types of work resources. Based on careful and mindful research design, they found that social support at work positively influenced the development of PsyCap, while PsyCap and crafting for social job resources were negatively related. This interesting, if not paradoxical, finding stimulate our reflection for the substitution/complementarity effects of strategic work resources, especially when in the context of workplace requiring creative, innovative, and entrepreneurial contributions. This study offered guidance for proper usage of individual/collective resources in workplace.

Tang et al. examined and explained why and how the PsyCap could lead to employee innovative behaviors, with the answer of the mediating mechanisms of job satisfaction and organizational commitment. The results of Partial Least Square analyses demonstrated that companies wish to invest in PsyCap to increase employee innovative behaviors need to focus and take good care of employees' job satisfaction and then organizational commitment endorsed by PsyCap. Put differently, if job satisfaction and organizational commitment were not leveled up by PsyCap, the nutrition of PsyCap is aimless in terms of innovation conducts and goals. This (somehow *avant-garde*) argument implicated a strategic meaning of PsyCap beyond just a shared psychological asset of a collective.

Zhao et al.' work step into a higher education context to address the important research question: what are the influences of psychological capital (PC) on students' entrepreneurial intention (EI) in universities? Conceptually, the study distinguishes between the effects of traditional entrepreneurial capitals (i.e., financial, human, and social capitals) and that of PsyCap. Based on an analysis of 1914 university students, the study found that “… traditional capital is the direct factor to drive the behavior of entrepreneurship, while psychological factors do not directly affect EI, but improve EI by influencing traditional capital.” As the higher education systems around the world increasingly appreciate the values of entrepreneurship as a career option for graduate or to-be-graduate students, the results of this study is a good reference for potential entrepreneurs' self-examination of owned capitals, in order to prioritize resources commitment especially when (the truth is) it is nearly impossible to own all of the traditional capital and the psychological capital at the beginning of entrepreneurship. Academically, this study is comparable with the Wang et al. paper mentioned above, though conducted in different research contexts.

Another good work in higher education context is brought by Kong F. et al.. The paper investigates on the moderating effect of business role model and fear of failure on the relationship between entrepreneurial intention and behavior. Same with the study of Zhao et al., a large sample of 1865 students were surveyed. The results found that “(1) Entrepreneurial intention was positively influenced the entrepreneurial behavior; (2) Fear of failure weakened the relationship between entrepreneurial intention and action; (3) The moderating effect of business role model on entrepreneurial intention and behavior was confirmed.” The framework tested incorporated both positive (facilitator) and negative (impediment) factors in exploring the boundary condition of entrepreneurial intention and behavior. From intention to behavior, there is a gap that requires some intervening factors to function. The results of this study provided detailed information that entrepreneurship teachers or consultant can use to make sure that a want of a potential entrepreneur can turn into actions and an entrepreneurial dream can come into practice eventually.

The two perspective papers from Tang (a) and Tang (b) are reasonable and of conceptual references. The first paper discussed PsyCap of entrepreneurs and its influences on human resource development (HRD), while the second article discussed the role that PsyCap can play to facilitate entrepreneurial sustainability. Both topics require a full length article to discuss, though, what can be further expected, might be the relationships between PsyCap, HRD, and entrepreneurial sustainability. Either article put a lot of emphasis of the impacts from the PsyCap, from traditional Organization and Management perspectives. Enlightened by such word, we suggest that future works could do more beyond the much-discussed Strategic Management and individual-entrepreneur-focused rationales of Entrepreneurship as a research field, to more Organization Theory and Organizational Behavior related issues. Whereas, the former rationales might shed lights on the developmental aspects of entrepreneurship, the latter ones are vital in search of the answer for organizational sustainability of a new venture.

Based on social exchange theory and the resource-based view, Guo, Liu et al. talked about the joint influences of entrepreneurs' political skills and their social networks in the relationship between entrepreneurial psychological capital and new venture performance. Embedded in a politics logic, the authors utilized organizational theories well in motivating and explaining political skills as tactical capabilities and social network as relational power resources for entrepreneurs to achieve a bright success outcome of their new ventures based on the positive origin of their psychological capital. Most importantly, this article plays as a good example of re-create the meaning of important constructs in organizational studies in a specific context, here, of the social entrepreneurship. Exactly because the big differences between general and social entrepreneurship, the functioning factors of new venture success, no matter at what levels, would be heterogeneous and should be linked differently.

Similarly, Xu et al. offered an examination of important constructs re-connection into a meaningful conceptual model of empirical relationships in a volunteering context. Through mediation and moderation relationships, the constructs of PsyCap, role identification, perceived social support, organizational commitment, and volunteering are well-connected. The study was also conducted in a sound methodological base with over-a-thousand volunteers participated. This makes their results of good reference value, which was summarized in the Abstract. This study also told that in a context of social entrepreneurship like volunteering, the actions of volunteers are not simply stimulated by warm-hearted motivation, but are gradually formed via a (complex) function of a series of direct and indirect influence relationships between psychological factors.

Modesti et al. shared the interests and core spirit of this Research Topic by placing their stud in the social enterprises with a migratory background. Through a qualitative case study, they concentrated on the dynamic interplay of psychological and social capitals that might affect the operation of a social enterprise. The results suggested that the two important entrepreneurial capitals function independently in the initial stage but interact jointly in a later stage. Such finding makes this paper extremely important and interesting in that it reminds us that the functionality of different entrepreneurial capitals might occur sequentially (or even recursively we guess) in different combinations in different contexts.

## Concluding Remarks

To reflect on the Research Topic as a whole, we quote the important lines from the Frontiers' (publisher's) About Research Topic web page (https://www.frontiersin.org/about/research-topics), which stressed that guest editors of a RT should “… unite the world's leading experts around the hottest topics, stimulating collaboration and accelerating science” in an RT. Using this criterion, we find that there are more to do, even with the hard works that have been done by all authors in this RT. We did attract some of very interesting and thought-stimulating papers around the world, though, more related RT could be designed to do better in encouraging collaborations between scholars who had not been working or even knowing one another. And this could be done better with the participation of both established and cutting-edge groups of scholars in the field, in order to benefit inter-generational knowledge transfer and co-creation. Indeed, collaborative studies from diverse groups of researchers at different career stages can generate great impacts, since they share similar knowledge bases but simultaneously possess heterogeneous research interests, questions, skills, and resources that might generate even larger impact after re-combinations. The RT as a virtual platform might do well in collecting, reviewing, and editing articles to form a one-stop knowledge storage for readers. But it cost more to do exactly the same in forming collaboration in research. Sincerely, we wish this RT is just a first-step in our marches toward the goal of “accelerating science.” With more to be done, the very critical next step is the exchanges and collaborations of researchers of the different papers. In this way, a strong research community could grow itself sustainably.

## Author Contributions

All three guest associate editors contribute equally in editing this Research Topic. F-ST wrote the original draft of this article. KL and SS reviewed and revised it.

## Conflict of Interest

The authors declare that the research was conducted in the absence of any commercial or financial relationships that could be construed as a potential conflict of interest.

## Footnotes

^1^As much has been done by the articles in this Research Topic (RT) in citing important extant literature in the research field of psychological capital, we only cite (in this article) published papers in this RT. Please refer to the published paper in this RT for their good reviews of the literature.

